# The enhanced expression of genes encoding diguanylate cyclases under cold stress contributes to the adhesion and biofilm formation of *Shewanella putrefaciens* WS13

**DOI:** 10.3389/fnut.2022.1076932

**Published:** 2022-12-01

**Authors:** Ruheng Xiong, Jun Yan, Jun Mei, Jingxin Ye, Jing Xie

**Affiliations:** ^1^College of Food Science and Technology, Shanghai Ocean University, Shanghai, China; ^2^Laboratory for Quality and Safety Risk Assessment of Aquatic Products in Storage and Preservation of Ministry of Agriculture and Rural Affairs, Shanghai Ocean University, Shanghai, China; ^3^Shanghai Professional Technology Service Platform on Cold Chain Equipment Performance and Energy Saving Evaluation, Shanghai Ocean University, Shanghai, China; ^4^Shanghai Engineering Research Center of Aquatic Product Processing and Preservation, Shanghai Ocean University, Shanghai, China; ^5^National Experimental Teaching Demonstration Center for Food Science and Engineering, Shanghai Ocean University, Shanghai, China

**Keywords:** *Shewanella putrefaciens*, cold stress, c-di-GMP, adhesion, biofilm

## Abstract

*Shewanella putrefaciens* is a special spoilage bacterium of seafood during cold storage, which is easy to form biofilm and bring serious hazard to the seafood quality. Life cycle of biofilm starts after bacterial adhesion, which is essential for the formation and development of biofilm. As a ubiquitous second messenger in bacteria, c-di-GMP regulates the conversion between bacterial planktonic state and biofilm state. In this study, the adhesion and biofilm formation of *S. putrefaciens* WS13 under 4°C were compared to those under 30°C. Atom force microscope and scanning electron microscope were used to study the bacterial adhesion. Biofilm was analyzed by Fourier transform infrared spectroscopy, Bradford assay and phenol-sulfuric acid method. High-performance liquid chromatographic-tandem mass spectrometric and quantitative real-time PCR were applied to study c-di-GMP level and genes encoding diguanylate cyclases in cells, respectively. Results showed that the swarming mobility of *S. putrefaciens* WS13 was weaker under 4°C, however, the adhesive force under 4°C was 4–5 times higher than that under 30°C. Biofilm biomass, extracellular polysaccharides and extracellular proteins were 2.5 times, 3 times, and 1.6 times more than those under 30°C, respectively, but biofilm composition formed under both temperatures were similar. c-di-GMP level in *S. putrefaciens* WS13 under 30°C was no more than half of that in the corresponding growth stage under 4°C. Quantitative real-time PCR analysis also showed that the expression of genes encoding diguanylate cyclases were significantly enhanced under 4°C than that under 30°C. *S. putrefaciens* WS13 adapted to the cold stress by enhancing the expression of genes encoding diguanylate cyclases to promote bacterial adhesion and biofilm formation. This study provides a theoretical foundation for the research on the cold adaptation mechanism of specific spoilage bacteria of seafood based on c-di-GMP, and also provides a new idea to control seafood quality from the perspective of microbial molecular biology.

## Introduction

*Shewanella putrefaciens* is a specific Gram-negative bacterium in aquatic products such as *Litopenaeus vannamei* (1, 2), bigeye tuna (3), shellfish (4), and *Paralichthys olivaceus* (5). *S. putrefaciens* has strong biofilm-forming capability. The formation of biofilm enhances the antimicrobial resistance, antimicrobial tolerance, and stress tolerance of bacteria to strengthen their environmental tolerance, which is conducive to the survival of bacterial cells (6–8). Biofilm is a bacterial community that is formed when bacteria attach to biotic/abiotic surfaces, secret substances such as extracellular polysaccharides, extracellular proteins, extracellular DNA, and lipids, and encapsulate bacterial cells. Once formed, biofilm is difficult to remove (6, 9, 10). The formation of biofilm is divided into four stages: bacterial adhesion, bacterial aggregation, biofilm maturation, and detachment (11). Bacterial adhesion is the process by which bacteria in a planktonic state sense and adhere to biotic/abiotic surfaces. It has two stages, reversible adhesion and irreversible adhesion (12). At the stage of irreversible adhesion, bacteria cannot escape from biotic/abiotic surfaces, and irreversible adhesion starts the life cycle of biofilm formation. The adhesion of bacteria is the key to biofilm formation (12). Biofilm is the ubiquitous form of microbial cells in nature (13). The formation of biofilm is affected by factors such as temperature (14), metal ions (15), oxygen (16), and nutrients (17). A study on the mechanism of biofilm formation under environmental stress is helpful to control the hazard of biofilm.

To adapt to the complex and changeable environment, there are various signal transduction systems in bacterial cells to ensure the survival of bacteria, such as cyclic diguanylic acid (c-di-GMP) (18) and two-component systems (19). As the ubiquitous second messenger in bacteria, c-di-GMP modulates diverse biological phenotypes in bacteria such as virulence, flagellar motility, biofilm formation (17), and bacterial colonization (20). c-di-GMP was first discovered in *Gluconacetobacter xylinus* in 1987 (21), and its level in bacterial cells is regulated by diguanylate cyclases (DGCs) (22) and phosphodiesterases (PDEs) (22, 23). Most DGCs and PDEs have transmembrane helix structures which can sense various input signals such as oxygen, light, antibiotics (17), and temperature (24) to regulate the level of c-di-GMP. Previous studies (25, 26) have shown that the intracellular level of c-di-GMP can regulate the conversion between the planktonic state and biofilm state of bacteria. In addition, c-di-GMP can regulate the activity of flagella to control the movement ability of bacteria and then regulate bacterial adhesion, and also regulate the synthesis of extracellular polysaccharides and extracellular proteins to promote the formation of biofilm (25). However, few studies on the role of c-di-GMP in the formation of biofilm of specific spoilage organisms (SSOs) in seafood have been reported.

Based on the above, this study intended to reveal the correlation between c-di-GMP and the adhesion and biofilm formation of *S. putrefaciens* WS13 under cold stress. Our work showed the characteristic differences of adhesive force and biofilm of *S. putrefaciens* WS13 under 30 and 4°C and indicated the importance of c-di-GMP for bacterial adhesion and biofilm formation. This provides a theoretical basis for the study of c-di-GMP regulating the adhesion and biofilm formation of *S. putrefaciens* WS13 under cold stress.

## Materials and methods

### Strains and cultivation

*S. putrefaciens* WS13 strain, isolated from spoiled *Litopenaeus vannamei*, which was preserved by our group (NCBI No. CP028435.1) (27) was applied in this experiment. The strain was preserved in LB medium (Land Bridge Technology, Beijing, China) with 50% (v/v) glycerol (Sinopharm., AR, China) in a −80°C refrigerator and was activated to 8–9 log CFU/mL (OD_600_ value = 0.80) in fresh LB medium under 30°C for 150 rpm, and then, the culture was diluted at 0.1% ratio with LB medium for further use.

### Growth curve and swarming mobility of *S. putrefaciens* WS13

The diluted culture was cultivated under oscillating conditions (150 rpm) under 4 and 30°C, respectively, and 200 μl of culture was taken out aseptically and added into 96-well polystyrene microtiter plates to measure bacterial growth curves with BioTek Synergy 2 (Winooski, VT, United States) at 600 nm.

The swarming mobility of bacteria was measured as described (28) previously with slightly modified. The culture was cultivated in semi-solid LB media containing 0.25% agar under 30 and 4°C for various times (12, 24, 48, 72, 96, 120, 144, and 168 h under 4 °C; 8, 12, 24, 36, 48, 60, 72, and 84 h under 30 °C), respectively. The sizes of bacterial colonies in the experiment were observed to analyze the swarming mobility of bacteria.

### Atom force microscope

The adhesive force of *S. putrefaciens* WS13 was measured by AFM (Bioscope resolve, Bruker, Germany) as described (29).

### Cell preparation

The diluted culture was cultivated in LB medium under 4 and 30°C to 8–9 log CFU/mL (OD_600_ value = 0.80), respectively. After centrifugation (9500 rpm, 8 min, 4°C), harvested cells were treated with 0.01 M sterile phosphate-buffered saline (PBS, pH 7.0) three times and suspended in Milli-Q grade water (OD_600_ value = 0.20–0.40). The suspension was applied to prepare cell probes (To minimize viability loss, cells could be treated with 2.5% (w/v) glutaraldehyde precooled under 4°C in advance and kept under 4°C).

### Probe design and detection

The suspension prepared was set on the glass slide at room temperature and connected to polystyrene plastic spheres (PS spheres, diameter, 12 μm) by electrostatic attraction for 20 min. Bacteria-PS spheres prepared were tenderly cleaned with Milli-Q grade water to divide unattached cells. A biological microscope (DM1000 LED, Leica, Germany) at a magnification rate of 900X was used to ensure that cells were fixed on PS spheres. PS spheres with cells were dispersed on the carrier chip and bonded to the MLCT-F tipless cantilever of AFM with AB glue. The experiment which applied the system of Dimension Icon (Bruker, Santa Barbara, CA) was operated at room temperature. First, the polystyrene plastic (PS) substrate was probed with a bare probe to set a baseline for comparing the force measurements with the designed probe. The elastic constant of the probe cantilever was 0.1 N/m. Spring constants of the tips were in the range of 0.6 N/m and were measured for each probe. The loading force was set to 1 nN. The adhesive force was measured by the formula F = k^*^sensitivity^*^deflection error. K and sensitivity of MLCT-F tipless cantilever were 0.6 N/m and 20 nm/v, respectively. Nanoscope 1.8 software was used to quantify the adhesive force that was obtained from the force–distance curves.

### Scanning electron microscope

According to bacterial growth curves, 1 mL of diluted culture was cultivated at 48-well polystyrene microtiter plates containing one piece of PS plastic sheet each under 30°C for 18 h and 4°C for 168 h, respectively. PS plastic sheets were taken out, washed with 0.01 M sterile phosphate-buffered saline (PBS, pH 7.0, ACMEC biochemical, China) three times, and treated with precooled 2.5 % glutaraldehyde (ACMEC biochemical, China) under 4°C for 4 h. After dehydrated with gradient ethanol (30, 50, 70, 90, and 100 %, v/v) for 10 min each and dried naturally, plastic sheets were treated with gold plating. Scanning electron microscope (SEM, S4500; Hitachi, Tokyo, Japan) at 3 kV was applied to obtain bacterial adhesive state (50 nm objective multiple) and morphology (5 nm objective multiple).

### Analysis of biofilm

Biofilm biomass was measured quantitatively as described (6) previously with slightly modified. Briefly, 1 mL of diluted culture was added into 48-well polystyrene microtiter plates; then, the plates were covered by plastic to avoid evaporative loss. According to bacterial growth curves, after certain cultivation times (6, 12, 18, 24, 30, 36, and 42 h under 30°C; 24, 48, 72, 96, 120, 144, 168, 192, 216, 240, and 264 h under 4°C), biofilms in wells were carefully cleaned three times with 1 mL of 0.01 M sterile phosphate-buffered saline (PBS, pH 7.0) to remove unattached cells. After being dried for 25 min under 60°C, biofilms were stained with 1 mL of 0.2% (w/v) crystal violet (Sangon Biotech, Co., Ltd., Shanghai, China) for 15 min, then washed and dried as described above. Dye attached to the biofilm was released with 95% ethanol (v/v) for 10 min. Bio Tek Synergy 2 (Winooski, VT, United States) at 600 nm was applied to obtain biofilm content.

The components of biofilm were analyzed as described (30, 31) previously with slightly modified. The diluted culture was cultured at 30 and 4°C, respectively. According to bacterial growth curves, after certain cultivation times (10, 15, 20, 25, and 30 h under 30°C and 108, 132, 156, 180, and 204 h under 4°C), the culture was removed and biofilm was washed with 0.01 M sterile phosphate-buffered saline (PBS, pH 7.0) three times and was collected by scraping; then, the cells were transferred to 1 mL of 0.01 M KCl solution. After being treated with ultrasonic (20 kHz, five times, 10 s on/10 s off), the cells were filtered by 0.22 μm cellulose membrane (Sangon Biotech, Co., Ltd., Shanghai, China) and dried under vacuum (3–6 kPa) condition in the freeze dryer for 48 h to obtain anhydrous samples. The anhydrous samples were analyzed by Fourier transform infrared spectroscopy (FT-IR, Perkin-Elmer, Model 2000), Bradford assay, and phenol–sulfuric acid method to detect biofilm FT-IR characteristics, the content of extracellular proteins and extracellular polysaccharides, respectively. FT-IR studies were performed with up to 100 scans, a scan range of 4000 and 450 cm^–1^, and resolutions of4 cm^–1^.

### High-performance liquid chromatography-tandem mass spectrometry

Cyclic diguanylic acid was analyzed as described (32) previously with slightly modified. The diluted culture was cultivated in LB medium under 30 and 4°C, respectively. According to bacterial growth curves, an equal volume of solution was extracted at the lag phase, early logarithmic phase (early log), middle logarithmic phase (mid-log), late logarithmic phase (late log), and stationary phase, respectively. Formaldehyde at a final concentration of 0.19% was added. After centrifugation (9500 rpm, 8 min, 4°C), harvested cells were treated with iced deionized water, centrifuged as described above, and then resuspended in 0.5 mL of iced deionized water to be heated at 96°C for 5 min. After adding 0.925 mL of ice anhydrous ethanol each, solutions were treated with vortex condition (30 s), centrifugation (13000 rpm, 4 min, 4°C), freeze-drying, and 50% (v/v) methanol to create resuspension solution. The samples were filtered by 0.22 μm cellulose membrane and analyzed by HPLC-MS-MS to quantify the intracellular c-di-GMP level.

### Quantitative real-time PCR analysis

The diluted culture, under 4 and 30°C at 8–9 log CFU/mL (OD_600_ value = 0.80), respectively, was centrifuged (10000 rpm, 5 min, 4°C) to extract RNA. The RNA was obtained with the RNA extraction kit (ACMEC Biochemical Co., Ltd., Shanghai), and its concentration was evaluated with an ELISA reader (Labsystems, Multiskan EX). The prepared RNAs were reverse-transcribed into cDNA following M-MLV 4 First-Strand cDNA Synthesis Kit (Biomed, Beijing) which were then kept at – 20°C.

Quantitative real-time PCR was carried out with the QuantStudio™ 1 Real-Time PCR System (Thermo Fisher Scientific, Waltham, MA USA). The reaction system consisted of 10 μl of 2X SYBR Green qPCR Master Mix, 0.4 μl of 10 μM F Primer, 0.4 μl of 10 μM R Primer, 7.2 μl of dd H_2_O, and 2 μl of cDNA. The reaction system without cDNA served as a negative control. 16S rRNA was applied as the reference gene, and the gene-specific primers were designed and synthesized by Sangon (Sangon, Biotech Co., Ltd, Shanghai, China). The primers used in this experiment for quantitative real-time PCR are shown in Table 1.

**Table 1:** Primer sequences of the quantitative real-time PCR assay.

**Gene name**	**Forward primer (5′-3′)**	**Reverse primer (5′-3′)**
16s rRNA	ACTCCTACGGGAGGCAGCAG,	ATTACCGCGGCTGCTGG
AVV82427.1	TTACTGAACGAGGAAATCACTACG	AACTACAACGCCAAGCAAGC
AVV82583.1	TAATATCAGTGCCTCTTTTGGTGT	GAGTCGTTTGGCTTCATAGAGTAA
AVV82979.1	CAACATAAAGAAGAAAACCAGAAGC	CCACATCAATAATCACCACGC
AVV83156.1	GTTGAGCCGCTTGTTGTCC	GAGCTGCTTCCATGTCATATTTAG
AVV84237.1	TGGTGATGCCTATGATGGTTC	AATGCCGCACTCGGACTC
AVV84424.1	AAATCCTGTCTGGGTCTCGC	ACCGAGTAAGTCTCCCATCCA
AVV84755.1	ATCCAATCATCTGGGCGTAA	TAGTATTTCTTTGGGAATGTGAGC
AVV85279.1	TACCTCTTTTAGCATTACCCTCCT	CGCCGACTATAGCTGACTTCTT
AVV85746.1	GCAAAAGATTGAGCGTCGG	TTTACCCATTCCAGCAGGC
AVV86051.1	TCTATTTGGGAGCGGGAAC	GCCCCTGGGTAAGAATAAAGA

Quantitative real-time PCR was operated with the following conditions: 50°C for 2 min; 95°C for 10 min; 40 cycles of 95°C for 15 s; and 60°C for 1 min. Genes were selected (27), and the expression level of genes was analyzed by the 2^−ΔΔCT^ method in triplicate. Statistical analysis was performed by Origin Pro 2021, and error bars represent the SD from the mean of measurements.

### Data analysis

The experiments were carried out at least three times independently. Analysis of microbiological results was converted to OD and log CFU/mL. Origin Pro 2021 (Origin Lab Corp., Northampton, MA, United States) was applied to analyze the content of biofilm, biofilm components, and other data obtained. SigmaStat 3.5 was applied to analyze bacterial adhesive force. Differences at *p*-value <0.05 were regarded as statistically significant.

## Results

### Growth and swarming mobility of *S. putrefaciens* WS13

**Figure 1:**
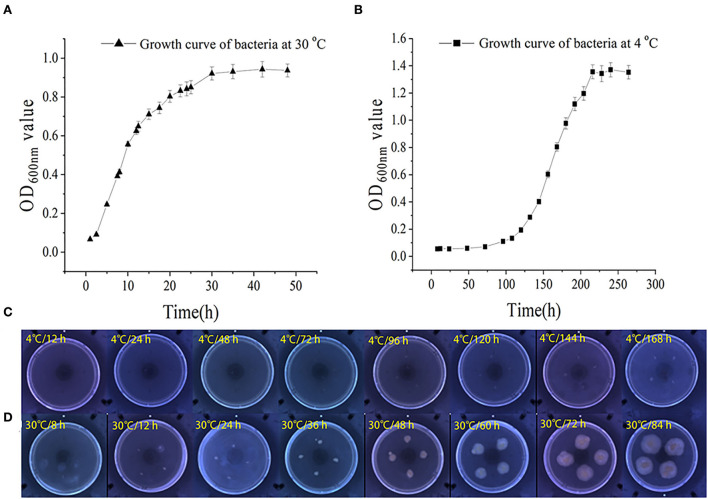
Growth and swarming mobility of *S. putrefaciens* WS13. **(A)** Growth curve of *S. putrefaciens* WS13 under 30°C. **(B)** Growth curve of *S. putrefaciens* WS13 under 4°C. **(C)** Swarming mobility of *S. putrefaciens* WS13 under 4°C. **(D)** Swarming mobility of *S. putrefaciens* WS13 under 30°C.

The growth and swarming mobility of *S. putrefaciens* WS13 are shown in Figure 1. The results showed that the lag phase under 30°C was < 2.5 h, and the lag phase under 4°C was significantly longer than 50 h. The maximum density value of cells under 4°C was higher than that under 30°C. At the stationary phase, the cell density of bacteria under 30°C was 3–5 log CFU/mL inferior to that under 4°C. The results of bacterial growth curves indicated that cold stress slowed down the bacterial growth rate and raised its density.

With the prolongation of incubation time, the sizes of colonies cultured under 4°C did not change significantly. However, the colonies cultured under 30°C appeared earlier, and the sizes of colonies increased with the extension of culture time. It showed that cold stress weakened bacterial swarming mobility (33) and made bacteria swarm in the smaller zone to form smaller colonies.

### Atom force microscope

**Figure 2:**
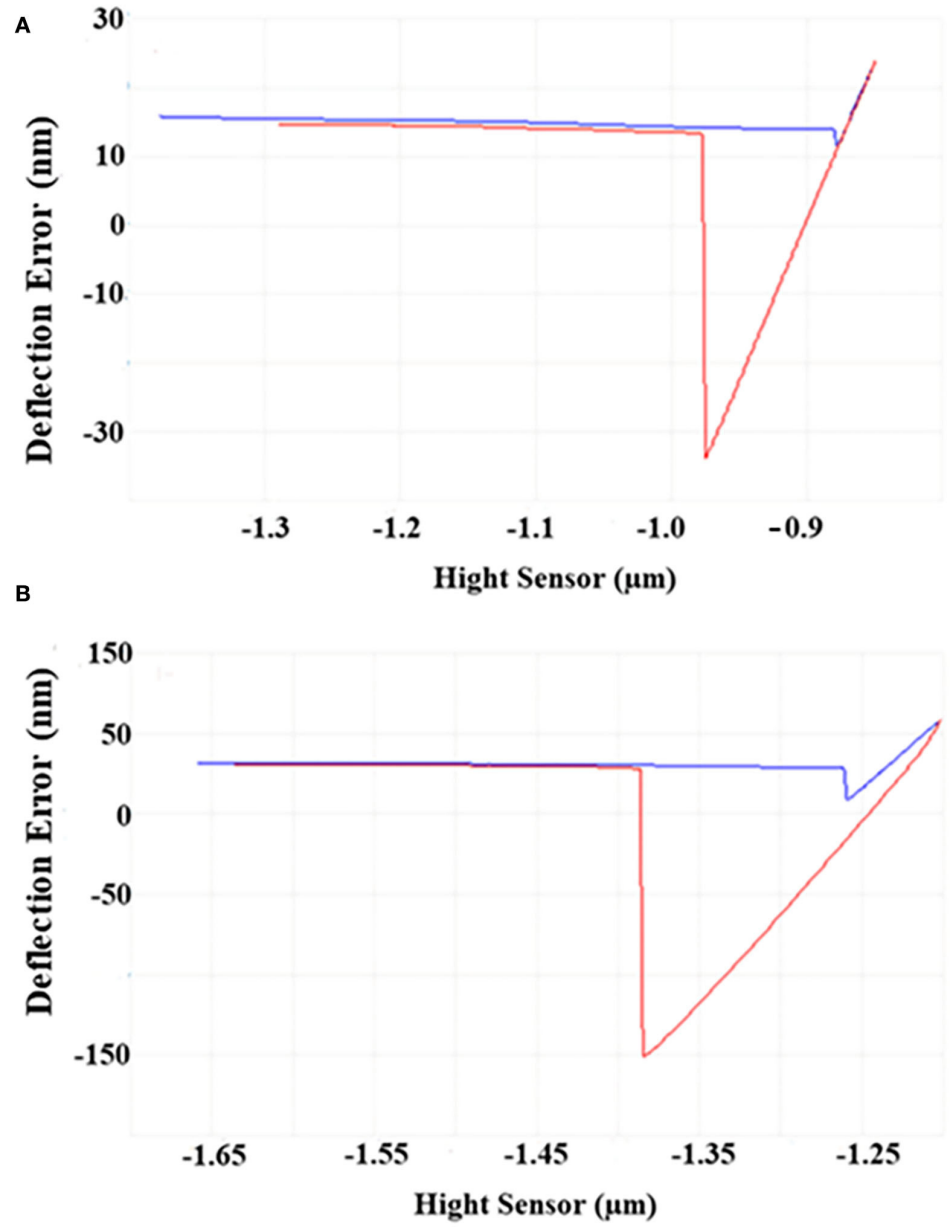
AFM images of *S. putrefaciens* WS13. **(A)** AFM image of *S. putrefaciens* WS13 under 30°C. **(B)** AFM image of *S. putrefaciens* WS13 under 4°C.

As shown in Figure 2, the horizontal baseline measured by the bare probe and the approach–retraction curve measured by sample probes were obtained. After calculation, the maximum difference between the two curves represented the adhesive force of the sample to the matrix. Under 4°C, the adhesive force of cells was four–five times higher than that under 30°C, demonstrating that the adhesive force of *S. putrefaciens* WS13 under 4°C was stronger than that under 30°C.

### Scanning electron microscope

**Figure 3:**
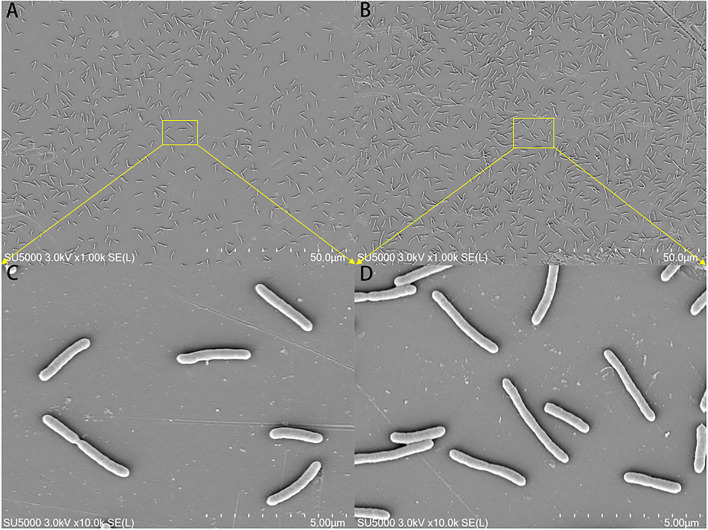
SEM images of the mature biofilm of *S. putrefaciens* WS13 under 30 and 4°C, respectively. The scale bar in **(A,B)** represents 50.0 μm. The scale bar in **(C,D)** represents 5.00 μm.

The morphological changes in the mature biofilm of *S. putrefaciens* WS13 under 30 and 4°C are shown in Figure 3. Cells in yellow rectangles in SEM images A and B were amplified as presented in C and D. SEM images indicated that the number of cells under 4°C was more than that under 30°C in matured biofilm. Bacterial morphology was affected by temperature, but the affection was tiny.

### Analysis of biofilm

**Figure 4:**
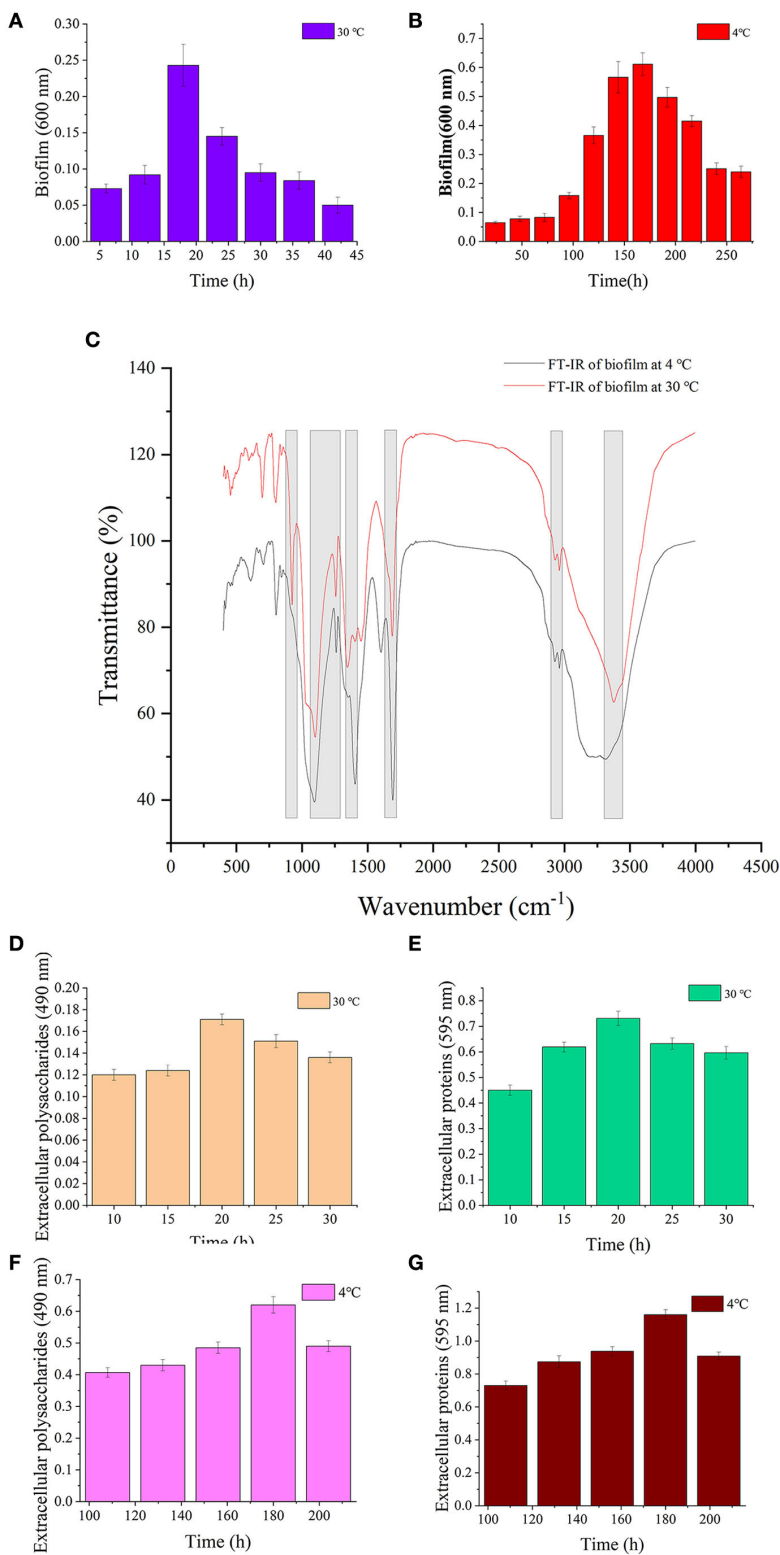
Biofilm analysis of *S. putrefaciens* WS13. **(A)** Biofilm biomass formed under 30°C. **(B)** Biofilm biomass formed under 4°C. **(C)** FT-IR spectra of *S. putrefaciens* WS13 under 30°C (red line) and 4°C (black line). **(D)** The extracellular polysaccharides content of EPS under 30°C. **(E)** Extracellular proteins of EPS content under 30°C. **(F)** The extracellular polysaccharides content of EPS under 4°C. **(G)** Extracellular proteins of EPS content under 4°C.

The results of biofilm biomass and components are shown in Figure 4. The biofilm matured under 30 and 4°C at 18 and 168 h, respectively (Figures 4A,B). *S. putrefaciens* WS13 produced more biofilm under cold stress. The results of Fourier transform infrared spectroscopy (FT-IR) characteristics of the mature biofilm formed under 30°C at 18 h and 4°C at 168 h are shown in Figure 4C, respectively. The characteristic wave numbers are shown in Table 2. Bands of extracellular polysaccharides were at 920 cm^−1^, 1100–1260 cm^−1^, and 3300–3400 cm^−1^, respectively. The peak at 1450 cm^−1^, 1690–1700 cm^−1^, and 3300–3400 cm^−1^ indicated the existence of extracellular proteins in biofilm, respectively. Other biomacromolecules such as lipids were verified in the biofilm, which is consistent with the previous studies (30, 34). FT-IR spectra of biofilm formed under 30 and 4°C were roughly the same. Extracellular polysaccharides and extracellular proteins reached the maximum value at 20 h and 180 h under 30 and 4°C, respectively. The maximum value of extracellular polysaccharides and extracellular proteins under 4°C was three and 1.5 times higher than that under 30°C, respectively.

**Table 2:** Assignment of the main bands of FT-IR spectra.

**Wavenumber (cm^−1^)**	**Type of Vibration**	**Assignment**	**References**
3300–3400	ν (N-H), ν (O-H)	carbohydrates, amide I	(30, 34)
2900–2960	ν (C-H)	lipids	
1690–1700	ν (C = O), ν (C-N) or τ (HOH)	amide I	
1400–1450	τ (C-H), ν (C = O) or τ (COO)	proteins, lipids	
1100–1260	ν (C-OH), ν (C-O-C) and P bonds in saccharide region	carbohydrates	
920	ν (O-H)	carbohydrates	

*Type of vibrations: (ν) stretching and (τ) bending.

### High-performance liquid chromatography-tandem mass spectrometry

**Figure 5:**
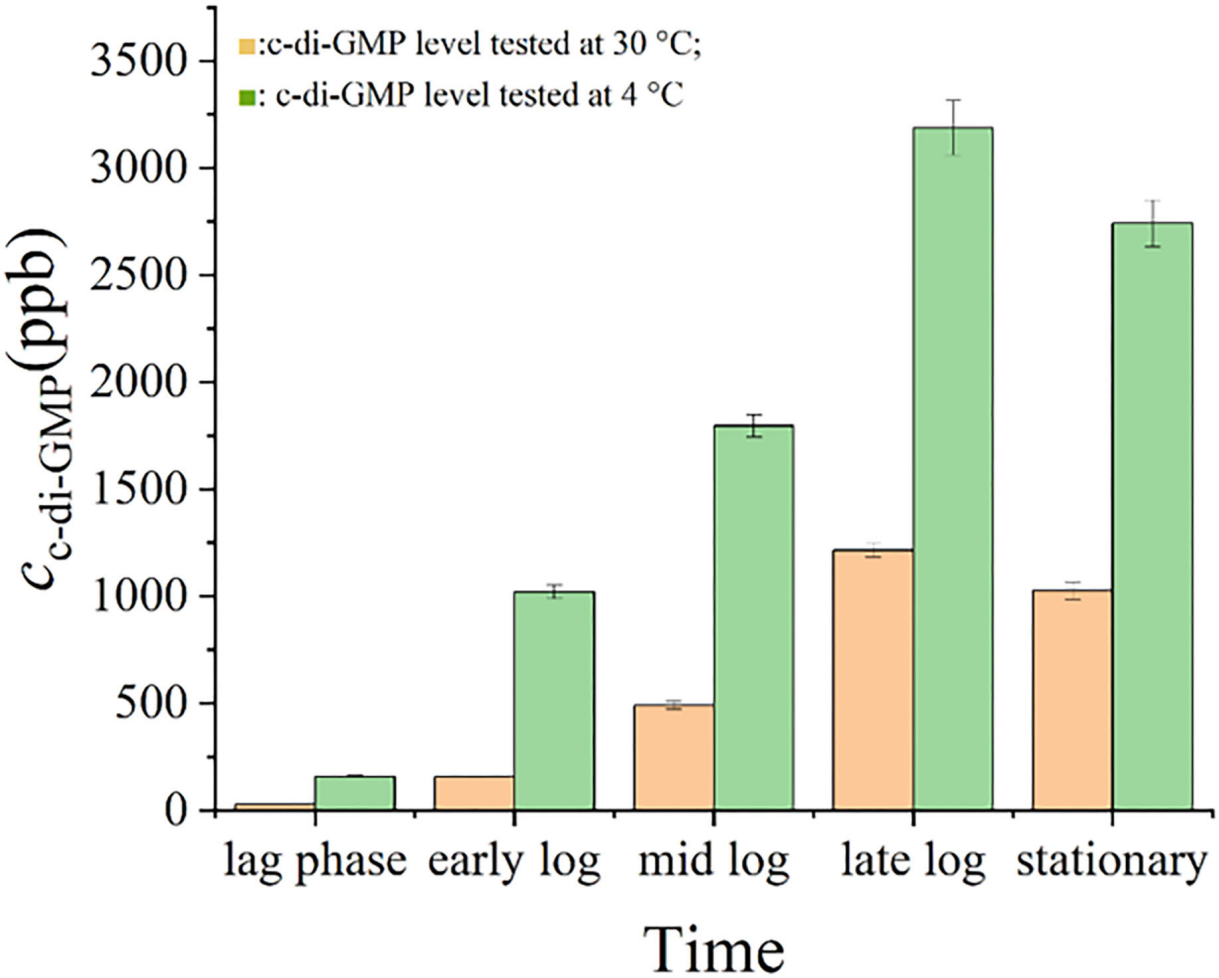
c-di-GMP levels in *S. putrefaciens* WS13. c-di-GMP levels in *S. putrefaciens* WS13 under 30 (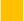) and 4°C (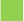).

Figure 5 shows that the intracellular levels of c-di-GMP in *S. putrefaciens* WS13 varied at different growth stages under 30 and 4°C. The level of c-di-GMP in bacterial cells increased first and then decreased both under 30 and 4°C. Mostly, the c-di-GMP level in cells was higher under 4°C than that under 30°C. Under the same culture stage, the intracellular c-di-GMP level under 30°C was < 50 % of that under 4°C.

### Quantitative real-time PCR analysis

**Figure 6:**
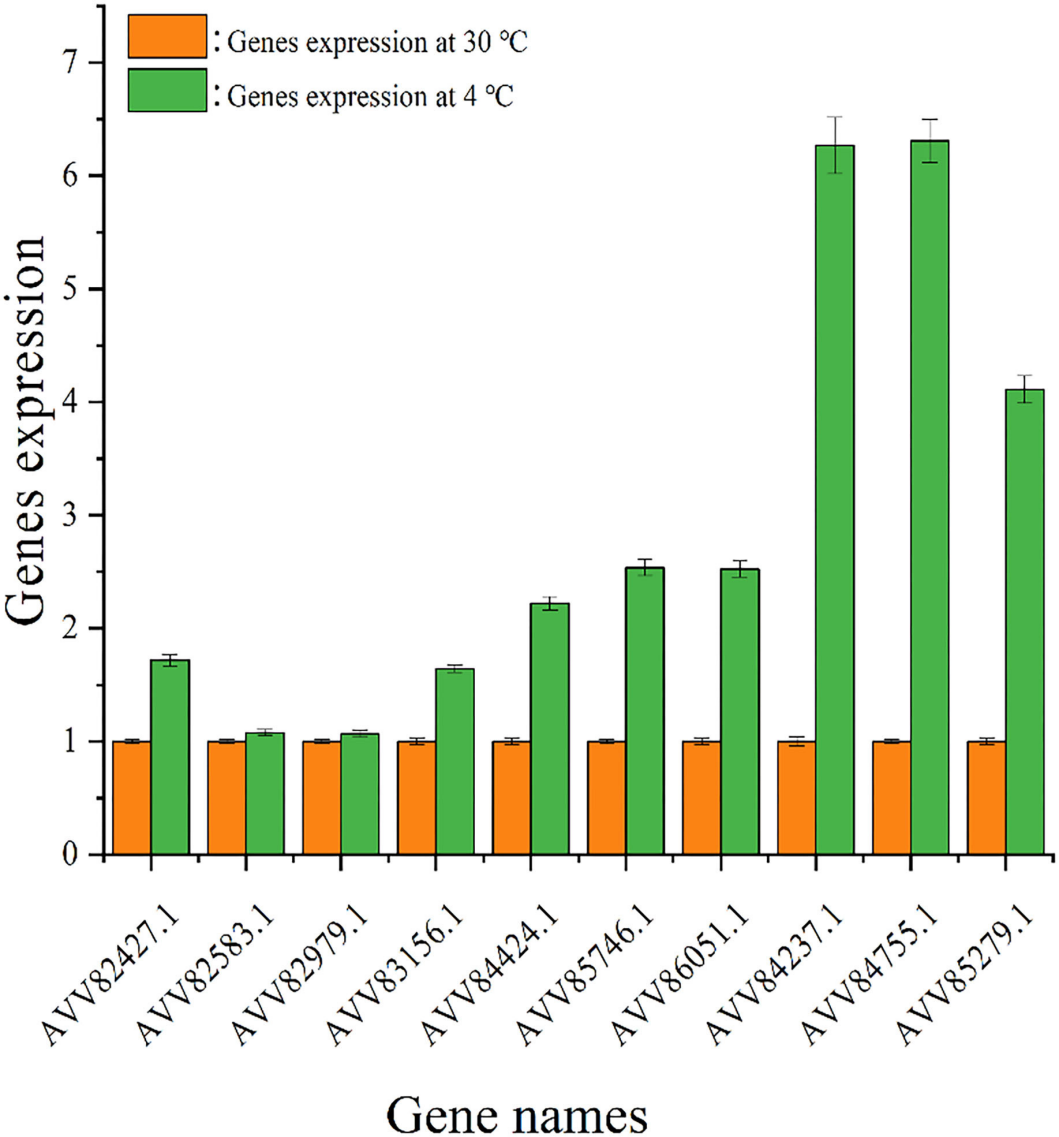
Expression of genes encoding diguanylate cyclases. The expression of genes encoding diguanylate cyclases under 30 (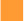) and 4°C (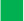).

The impact of temperature on the expression level of genes encoding diguanylate cyclases was evaluated by quantitative real-time PCR analysis, and the result is shown in Figure 6. Under 4°C, the expression levels of all tested genes were higher than those under 30°C, and the change in gene expression under cold stress was different. The expression levels of genes AVV82427.1, AVV82583.1, AVV82979.1, and AVV83156.1 under 4°C were upregulated by < 2 times (1.72, 1.08, 1.07, and 1.64, respectively) than that under 30°C, and the expression levels of genes AVV84424.1, AVV85746.1, AVV86051.1, AVV84237.1, AVV84755.1, and AVV85279.1 were upregulated much more (2.22, 2.54, 2.52, 6.27, 6.31, and 4.11,respectively).

## Discussion

*S. putrefaciens* WS13 is a specific spoilage organism (SSO) of seafood under cold storage and is related to aquatic product disease named *shewanellosis* (35). *S. putrefaciens* has a strong biofilm-forming capability. Our group has found that the formation of biofilm of *S. putrefaciens* WS13 is conducive to its survival under cold stress (6, 36). Further study found that genes such as *motA, ppsA*, and *fabs* family expressed in *S. putrefaciens* WS13 under 4°C help attenuate bacterial motility, enhance bacterial adhesion, and increase biofilm fluidity (36, 37), which was beneficial for bacteria to form biofilm under cold stress. Biofilm formation began after bacteria adhered to the surface of the object, and the adhesion of bacteria was regulated by c-di-GMP (11, 25). However, the characteristics of adhesion and biofilm of *S. putrefaciens* WS13 under cold stress, as well as intracellular c-di-GMP level, which regulated these two, needed further study. Therefore, the refrigeration temperature (4°C) and an optimum growth temperature (30°C) were selected in this work to investigate the difference between those features of *S. putrefaciens* WS13.

The swarming mobility, intracellular c-di-GMP level, biofilm, cell morphology, and adhesion, as well as gene expression of *S. putrefaciens* WS13 under 30 and 4°C, were investigated in this work. *S. putrefaciens* WS13 grew slower under 4°C, but its final density was higher than that under 30°C. The swarming mobility of *S. putrefaciens* WS13 under 4°C was weaker than that under 30°C. Studies on *Listeria monocytogenes* and *Ralstonia solanacearum* strains showed that bacteria under cold stress achieved a greater proliferation rate and a lower growth rate, and bacterial mobility on growing colonies was attenuated (38, 39), which were consistent with our work. Researchers have reported that the c-di-GMP level could affect bacterial swarming mobility through flagella (40) or fimbriae (41), and bacterial swarming mobility affected bacterial self-aggregation (42), which in turn affected bacterial biofilm formation (43). The high c-di-GMP level in bacterial cells would bind the proteins such as YcgR to affect the flagellar motor, weakening bacterial swarming mobility, which improved bacterial self-aggregation. Those results would promote biofilm formation too (43, 44). Our work found that the intracellular c-di-GMP level in *S. putrefaciens* WS13 increased and weakened the ability of swarming mobility of *S. putrefaciens* WS13 under cold stress compared to that under 30°C, which was consistent with the previous study.

Bacterial adhesion is affected by many factors such as cellular appendages and extracellular substances (45–47). Our work found that *S. putrefaciens* WS13 cells had stronger adhesive force under 4°C than that under 30°C. The biomass of EPS of *S. putrefaciens* WS13 under 4°C was higher, and extracellular proteins and extracellular polysaccharides were higher than those that synthesized under 30°C. According to the research, EPS affected the adhesion of bacteria (48–50). This might indicate that *S. putrefaciens* WS13 would strengthen bacterial adhesion by increasing the content of EPS under cold stress. However, the details for those remained for further analysis.

The content of EPS and its components (such as extracellular proteins and extracellular polysaccharides) are regulated by many factors, such as c-di-GMP (51–53). The increase in the intracellular c-di-GMP level under cold stress may help to promote the synthesis and secretion of extracellular polysaccharides and extracellular proteins (51–53) and enhance bacterial adhesion, which is conducive for bacteria to survive under cold stress. It was found that the extracellular polysaccharides Pel and/or Psl contributed to the adhesion of *Pseudomonas Aeruginosa*, and the synthesis and secretion of Pel and/or Psl were regulated by c-di-GMP (25). In addition, it was found that c-di-GMP in *Pseudomonas putida* modulated the transcription factor such as FleQ, affecting the synthesis and secretion of adhesin LapA, which regulated the adhesion of *P. putida* (50). The increase in the intracellular c-di-GMP level under cold stress was identified to promote the synthesis and secretion of extracellular polysaccharides and proteins of EPS, which would strengthen bacterial adhesive force (51–53). This study also confirmed that c-di-GMP regulates extracellular polymers to affect the adhesion of bacteria.

Biofilm formed by *S. putrefaciens* WS13 under cold stress was also investigated. The results showed that the FT-IR characteristics, morphology, and structure of bacterial biofilm formed under 30 and 4°C were similar, which meant the composition of biofilm formed under both temperatures was almost the same. The intracellular c-di-GMP level affects biofilm formation (54). The high c-di-GMP level would increase bacterial extracellular matrix level and accelerate biofilm formation (55). Compared to that under 30°C, we found that the intracellular c-di-GMP level increased under cold stress, which was consistent with the conclusion that c-di-GMP regulated bacterial living lifestyle between biofilm state and planktonic state (56, 57). However, the effects of c-di-GMP under low temperature affecting bacterial biofilm composition and biofilm formation remained to be elucidated.

The level of c-di-GMP in bacterial cells affects the activity of proteases in the cytoplasm, which could hydrolyze the substances such as extracellular proteins that were located on the outer membrane of bacteria, changing the composition and structure of biofilm (58, 59). In *P. fluorescens Pf0-1*, the high level of c-di-GMP would bind to LapD and promote LapD to interact with the periplasmic protease LapG, which inhibited LapD to cleave the N-terminus of adhesin LapA and enhance the ability of bacterial adhesion (58, 59). Mostly, intracellular c-di-GMP at the high level would promote the secretion of EPS by controlling the regulatory factors to promote gene transcription. CuxR, the c-di-GMP-responsive protein in plant symbiotic α*-proteobacteria*, would stimulate the transcription of the gene cluster that synthesized EPS at high c-di-GMP levels, assisting bacterial adhesion (54). Also, researchers found that in *S. putrefaciens*, the high level of c-di-GMP could bind to the transcription regulator FlrA, which would strengthen the synthesis of adhesin BpfA, and reinforce bacterial adhesion (52). It explained the difference in FT-IR characteristics of bacterial biofilm formed under both temperatures. Biofilm biomass formed by *S. putrefaciens* WS13 under 4°C was higher than that under 30°C, which was consistent with our previous work (6). It demonstrated that cold stress promoted *S. putrefaciens* WS13 to form more biofilms. However, further work on how cold stress affected biofilm formation was needed. Our work found that under cold stress, the intracellular c-di-GMP level of *S. putrefaciens* WS13 was higher under 4°C than that under 30°C at the same growth stage, which affected the swarming mobility, biofilm, cell morphology, and adhesion of bacteria. The intracellular c-di-GMP level was regulated by DGCs and PDEs (22, 23), while environmental signals such as light, oxygen, (17) and temperature (24) would affect the expression of DGCs and PDEs and then regulate the level of c-di-GMP in cells. Quantitative real-time PCR analysis showed the expression of genes that encoded diguanylate cyclases was upregulated under cold stress, and the increased expression of genes contributed to the increase in c-di-GMP in bacterial cells. The high c-di-GMP level reduced bacterial swarming mobility, promoted bacterial adhesion and biofilm formation, and also promoted the bacterial ability to produce extracellular polysaccharides and extracellular proteins under 4°C, which directly improved bacterial adhesion, which made biofilm more than that under 30°C. This study evaluated the effects of c-di-GMP on the bacterial adhesion and biofilm formation of SSO in seafood under cold stress. However, the specific signal pathways of c-di-GMP regulating bacterial adhesion and biofilm formation need to be further studied. This study provides a theoretical basis for studying the effect of c-di-GMP on the adhesion and biofilm formation of *S. putrefaciens* WS13 under cold stress.

## Conclusion

This study analyzed the variation of c-di-GMP level and the expression of genes encoding diguanylate cyclases in *S. putrefaciens* WS13 under 30 and 4°C, as well as the effects of c-di-GMP on bacterial adhesion and biofilm formation. The expression of genes encoding diguanylate cyclases and the levels of c-di-GMP increased in *S. putrefaciens* WS13 under cold stress, which reduced the swarming ability of bacteria, enhanced the ability of cells to adhere, and promoted bacteria to form more biofilm compared to that under 30°C. In addition, the amount of EPS such as extracellular polysaccharides and extracellular proteins also increased, which also enhanced bacterial adhesion and biofilm biomass. This study provided valuable clues to better understand the effect of c-di-GMP on the bacterial adhesion and biofilm formation of *S. putrefaciens* WS13 under cold stress. Research on the mechanism of adhesion and biofilm formation of *S. putrefaciens* WS13 regulated by c-di-GMP under cold stress also laid a theoretical foundation for the development of technology to control the hazard caused by biofilm.

## Data availability statement

The datasets presented in this study can be found in online repositories. The names of the repository/repositories and accession number(s) can be found in the article/supplementary material.

## Author contributions

RX: data curation, writing—original draft preparation, writing—review and editing, and visualization. JYan: conceptualization, methodology, validation, investigation, resources, data curation, writing—review and editing, and visualization. JM and JYe: visualization. JX: conceptualization, methodology, validation, investigation, resources, data curation, writing—review and editing, visualization, supervision, project administration, and funding acquisition. All authors contributed to the article and approved the submitted version.

## Funding

This research was funded by the National Natural Science Foundation of China (No. 31972142) and the Science and Technology Commission of Shanghai Municipality (20DZ2292200 and 19DZ1207503).

## Conflict of interest

The authors declare that the research was conducted in the absence of any commercial or financial relationships that could be construed as a potential conflict of interest.

## Publisher's note

All claims expressed in this article are solely those of the authors and do not necessarily represent those of their affiliated organizations, or those of the publisher, the editors and the reviewers. Any product that may be evaluated in this article, or claim that may be made by its manufacturer, is not guaranteed or endorsed by the publisher.
